# Chloroplast Protein Degradation Pathways During Leaf Senescence: Coordination and Roles in Nitrogen Remobilization

**DOI:** 10.3390/plants15172576

**Published:** 2026-08-24

**Authors:** Dexing Jiang, Hui Xu

**Affiliations:** 1 College of Life Sciences and Chemical Engineering, Jiangsu Second Normal University, Nanjing 211200, China; 2 College of Agricultural and Forestry Engineering and Planning, Tongren University, Tongren 554300, China

**Keywords:** leaf senescence, chloroplast degradation, ubiquitin–proteasome system, nitrogen remobilization, autophagy, TOR-SnRK1 signaling

## Abstract

Leaf senescence is a highly programmed developmental process that enables nutrient remobilization from source to sink organs in plants. Chloroplasts serve as the primary nitrogen reservoir, and the timely degradation of their constituent proteins is essential for sustaining seed fill and crop yield. For efficient nitrogen recycling, the chloroplast proteome must be dismantled in a controlled manner through coordinated proteolytic pathways that function both inside and outside the organelle. Protein abundances are progressively reduced during senescence through the balanced action of degradation and the suppression of new synthesis, depending on the plant’s carbon–nitrogen status. Malfunctioning or damaged chloroplast components are selectively eliminated. To date, three major classes of chloroplast protein degradation systems have been characterized: the cytoplasmic ubiquitin–proteasome system (UPS), vacuolar degradation pathways, and intra-chloroplast proteases. Here we provide an updated, comprehensive overview of the three chloroplast protein degradation machineries and discuss their coordinated roles in nitrogen remobilization. We also consider how these pathways might be manipulated to improve nitrogen use efficiency (NUE) and crop productivity.

## 1. Introduction

Senescence, triggered by chronological age or environmental stress, represents a genetically programmed terminal phase that orchestrates the active remobilization of nutrients from source to sink tissues [1]. During long-term evolution, plants have developed a highly ordered programmed cell death (PCD) mechanism controlled by senescence-associated genes (SAGs), which eliminates inefficient and aged photosynthetic organs to cope with frequent nutrient deprivation stress [2,3]. The most visible hallmark of leaf senescence is the degradation of chlorophyll and chloroplasts [4]. This process enables the recycling and redistribution of nutrients from source organs to sink organs and is crucial for grain filling and biomass accumulation [5,6]. In agricultural production, senescence-related traits such as stay-green and delayed senescence are closely linked to crop yield and stress tolerance [1,7]. This review primarily focuses on developmental leaf senescence, while drawing extensively on mechanistic insights from stress-induced senescence models, which share core molecular components with developmental senescence.

Leaf senescence directly affects source–sink relationships, and the series of physiological events during leaf senescence are subject to strict temporal and coordinated regulation [5,8,9]. During leaf senescence, primary nitrogen assimilation capacity progressively declines, whereas the activities of key enzymes involved in nitrogen cycling and remobilization are significantly enhanced [8,10]. Moreover, multiple SAGs participate in nitrogen remobilization during leaf senescence [8]. Chloroplast proteins account for more than 70% of total leaf protein and are rapidly degraded during senescence [1]. In C_3_ plants, chloroplast nitrogen accounts for approximately 75 to 80% of total leaf nitrogen [11,12]. Therefore, the degradation of chloroplast protein components during senescence is the main source of nutrient recovery and reuse in plants, and also the largest contributor to seed nitrogen nutrition [3]. However, the mechanisms underlying chloroplast protein degradation during senescence are not yet fully understood [13]. Elucidating the molecular pathways and regulation of chloroplast protein degradation is particularly important for understanding plant nitrogen remobilization.

Chloroplast proteins can be classified into three types based on their localization, namely envelope proteins, stromal proteins and thylakoid proteins [14], constituting the main storage form of nitrogen in plants. Among them, ribulose-1,5-bisphosphate carboxylase/oxygenase (Rubisco) is the most abundant stromal protein in the chloroplast. In C_3_ plants, Rubisco accounts for approximately 50% of soluble leaf protein and 20–30% of total nitrogen [15,16]. To date, three major classes of chloroplast protein degradation systems have been characterized: the cytoplasmic ubiquitin–proteasome system (UPS), which specifically degrades short-lived, regulatory or structurally abnormal proteins [17,18]; vacuolar degradation pathways (including both autophagy-dependent and autophagy-independent routes), which can perform bulk degradation of long-lived proteins or entire organelles such as damaged chloroplasts [19]; and the intra-chloroplast protease system, which is responsible for chloroplast protein turnover and quality control [20,21]. Thus, chloroplast protein degradation during leaf senescence is not accomplished by a single pathway, but rather by a complex network coordinated by the UPS, the autophagy system, and the chloroplast internal protease system. In this review, we attempt to summarize recent advances in our understanding of the regulatory mechanisms of these three chloroplast protein degradation systems and their contributions to nitrogen remobilization in senescent leaves.

## 2. UPS-Mediated Chloroplast Protein Degradation

**Figure 1:**
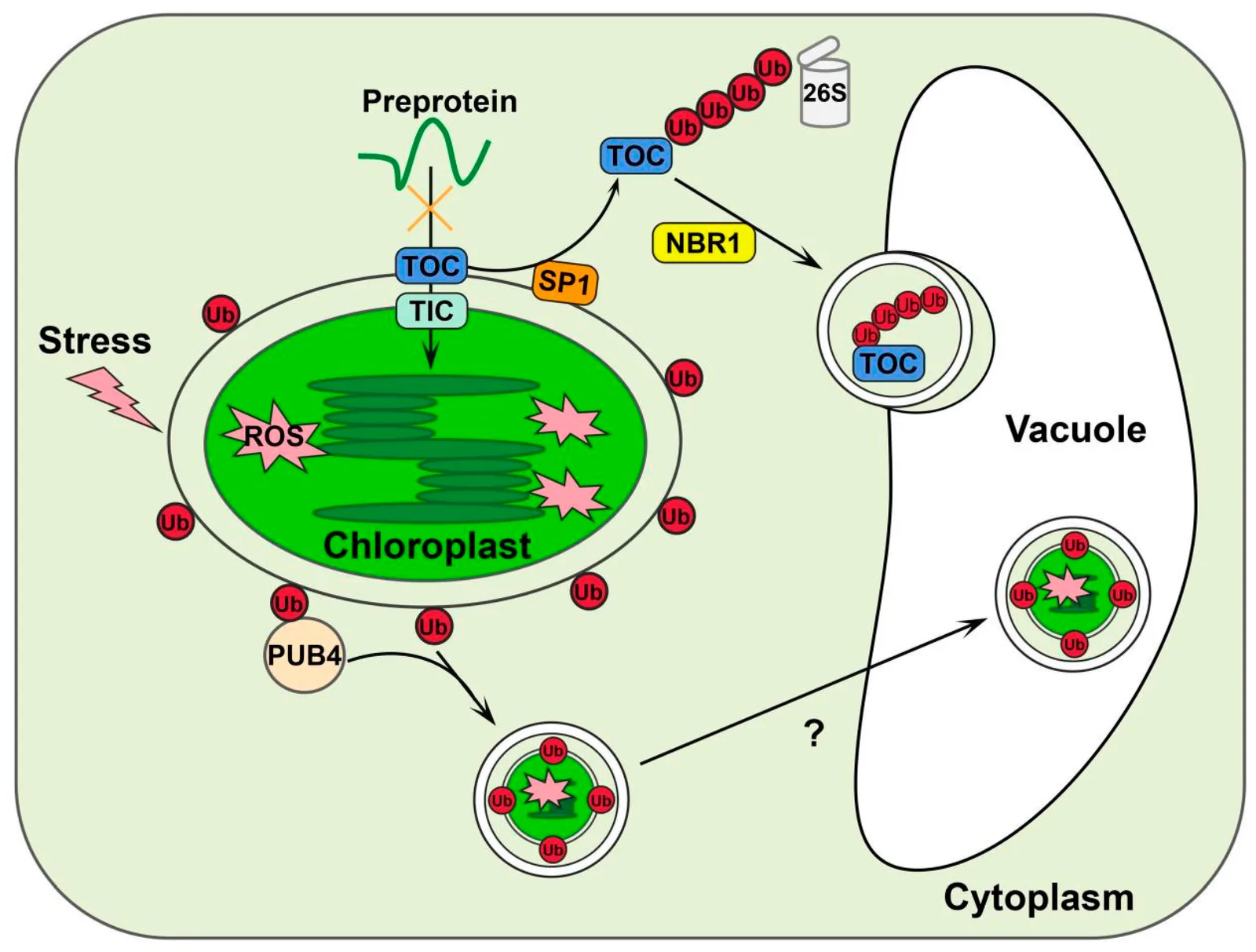
Schematic model for UPS-mediated chloroplast degradation. Stress induces excessive production of ROS, which correlates with the activation of the chloroplast outer membrane-localized E3 ligase SUPPRESSOR OF PPI1 LOCUS1 (SP1), thereby mediating the specific degradation of ubiquitinated TOC components by the 26S proteasome. This reduces the import of chloroplast precursor proteins and inhibits photosynthesis, thereby preventing further ROS production and chloroplast damage. Additionally, TOC components are also subject to K63-linked polyubiquitination and selective autophagy, with NBR1 mediating the degradation of ubiquitinated TOC components via macroautophagy. When chloroplasts over-accumulate singlet oxygen (^1^O_2_), the cytoplasmic E3 ligase PLANT U-BOX 4 (PUB4) may broadly ubiquitinate chloroplast outer membrane proteins, subsequently triggering vacuole-dependent clearance of entire damaged chloroplasts through an unknown mechanism.

The UPS is a rapid regulatory mechanism for selective protein degradation in plants and plays critical roles in plant growth and development, hormone signaling, abiotic stress responses, embryogenesis and senescence [22,23]. As a specific protein degradation mechanism, the UPS process includes ubiquitination of the target protein, recognition by the 26S proteasome, and eventual degradation within the proteasome. Ubiquitination is a complex process that relies on a cascade of E1 activating enzymes, E2 conjugating enzymes and E3 ligases, with the diversity of E3 ligases significantly expanding the substrate specificity of the UPS [24]. Two ubiquitin-dependent chloroplast protein degradation pathways mediated by the ubiquitin E3 ligases SUPPRESSOR OF PPI1 LOCUS1 (SP1) and PLANT U-BOX 4 (PUB4) have recently been identified (Figure 1).

### 2.1. SP1

Most chloroplast precursor proteins are synthesized in the cytoplasm and imported into the chloroplast stroma via translocon complexes in the outer and inner envelope membranes (the TOC and TIC complexes, respectively) [25,26]. Direct evidence for ubiquitination-dependent degradation of outer envelope proteins came from the discovery of SP1 located in the chloroplast outer envelope membrane [27]. Studies in Arabidopsis *sp1* mutants and overexpressing lines showed that SP1 levels are negatively correlated with TOC protein abundance, and that SP1 directly interacts with various TOC proteins, mediates their ubiquitination, and then targets them for degradation by the cytoplasmic 26S proteasome [27]. SP1-mediated ubiquitination and degradation of TOC proteins regulate the import of chloroplast precursor proteins, thereby suppressing photosynthesis under stress and reducing the risk of excessive ROS production and photo-oxidative damage [28].

Since TOC proteins are integral membrane components, ubiquitinated TOC proteins must overcome the physical and energetic barriers to detach from the chloroplast outer envelope membrane before their degradation in the cytosol. Recently, SP1 was shown to function in a novel pathway termed CHLORAD (chloroplast-associated protein degradation), in which SP2 and CDC48 cooperate to bring about retrotranslocation of SP1-ubiquitinated TOC proteins from the outer envelope membrane to the cytoplasm for degradation by the 26S proteasome [18,29]. In addition to SP1, two homologous E3 ligases, SP1-like1 (SPL1) and SPL2, also localize to the chloroplast outer envelope membrane but antagonistically regulate CHLORAD [30]. SPL1 negatively modulates SP1-mediated ubiquitination, whereas SPL2 shares partial functional redundancy with SP1. Accordingly, *SPL2* loss delays senescence while its overexpression accelerates it, and *SPL1* loss accelerates senescence. Another study found that Arabidopsis *sp1* mutants undergo normal chlorophagy under high light, indicating that SP1 is not involved in chlorophagy [31]. In addition to being regulated by the UPS, TOC components are also subject to K63-linked polyubiquitination and selective autophagy [32]. As a selective autophagy adaptor protein, NBR1 targets TOC components (Toc33, Toc159 and Toc75) and mediates their relocalization to the vacuole for autophagic degradation [32]. Notably, this study revealed that K63-linked ubiquitin chains, rather than K48-linked chains, are preferentially recognized by NBR1, providing a molecular basis for the decision between the UPS (K48) and autophagy (K63). Thus, degradation of TOC proteins in the outer envelope may be modulated by two distinct pathways: SP1-mediated 26S proteasome pathway and NBR1-mediated autophagic pathway (Figure 1).

### 2.2. PUB4

In plants, ferrochelatase 1 and 2 (FC1, FC2) catalyze the formation of heme from protoporphyrin IX (Proto) and play important roles in chloroplast retrograde signaling [33]. Mutation of *FC2* disrupts the tetrapyrrole pathway, leading to excessive ROS production inside chloroplasts and triggering chloroplast degradation [34]. To identify mutations that could restore greening in the Arabidopsis *fc2* mutants, a second-site suppressor screen was performed in the *fc2* background and identified the *ferrochelatase two suppressor 29* (*fts29*) mutation (a missense mutation in *PUB4*). The *pub4* mutation suppressed the elevated chloroplast ubiquitination levels in the *fc2* mutant and inhibited chloroplast degradation [34]. Further analysis revealed that some damaged chloroplasts in the *fc2* mutant are interacting with globular vacuoles, whereas nearby organelles appear normal, suggesting that damaged chloroplasts might be selectively degraded. However, a recent study found that Arabidopsis *pub4* mutants undergo normal chlorophagy under high light, indicating that PUB4-mediated ubiquitination of chloroplast proteins is not essential for chlorophagy [31].

Cytoplasmic PUB4-mediated ubiquitination of chloroplast proteins and the subsequent triggering of chloroplast degradation represent a novel ubiquitin-mediated chloroplast quality control pathway. However, direct evidence that the ubiquitination substrates of PUB4 are outer envelope proteins is still lacking; it is only postulated that under oxidative stress, PUB4 selectively ubiquitinates damaged chloroplast proteins and drives their transport to the vacuole for degradation via an unknown pathway [35]. In addition, chloroplast thylakoid lumen and stromal proteins can be ubiquitinated and degraded by the 26S proteasome under oxidative stress, but the precise mechanism of intra-chloroplastic ubiquitination remains elusive [36,37].

## 3. Vacuolar Degradation Pathways of Chloroplast Proteins

**Figure 2:**
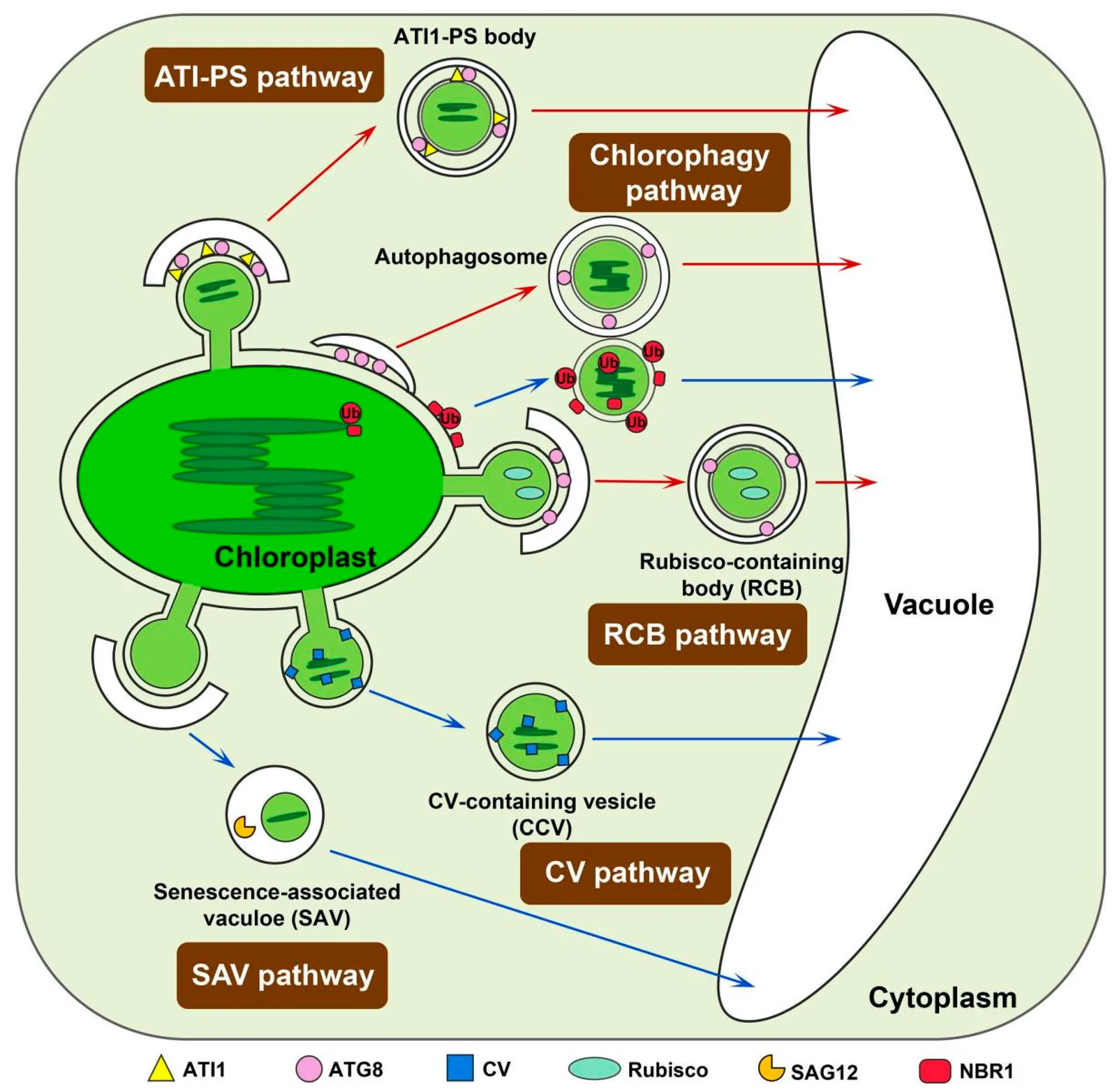
Pathways for vacuolar degradation of chloroplasts. These pathways can be either ATG-dependent (arrows in red) or ATG-independent (arrows in blue). In ATG-dependent (macroautophagy-like) pathways, Rubisco-containing body (RCB), ATI1-GFP-labelled plastid-associated body (ATI-PS), and entire chloroplasts are sequestered by ATG8 mediated autophagosomes and subsequently transported to vacuoles for degradation. Additionally, upon photodamage, NBR1 mediates the microautophagic delivery of entire chloroplasts to the vacuole. In ATG-independent (microautophagy-like) pathways, CV-containing vesicle (CCV) and senescence-associated vacuole (SAV) are degraded without the involvement of autophagosomes. ATG8, autophagy-related protein 8; ATI1, ATG8 interacting protein 1; SAG12, senescence-associated gene 12; NBR1, NEIGHBOR OF BRCA1 gene 1.

During leaf senescence or under environmental stress, chloroplast proteins need to be efficiently degraded and recycled. The vacuole, the largest hydrolytic compartment in plant cells, is a key site for chloroplast protein degradation and nitrogen recycling [1,6]. At least two major classes of mechanisms, autophagy-dependent and autophagy-independent pathways, are known to deliver chloroplast proteins to the vacuole for degradation (Figure 2) [1,38,39].

### 3.1. Autophagy-Dependent Degradation Pathways

Autophagy was first observed in the 1950s–1960s. From the 1990s onward, the Ohsumi laboratory identified more than 30 autophagy-related genes (*ATG*) in *Saccharomyces cerevisiae* through genetic screening, and their encoded products participate in various stages of autophagic vesicle formation, including induction, vesicle nucleation, expansion, maturation, and fusion with the vacuole [40]. In plants, autophagy typically occurs through the formation of double-membrane autophagosomes that enclose cargo (macroautophagy) or by direct engulfment of cargo by the vacuolar membrane (microautophagy) [38,41]. Although the molecular mechanism of microautophagy is not yet fully understood, macroautophagy involves more than 40 ATG proteins, and its activation is regulated by upstream phosphorylation modifications, ultimately leading to the formation of ATG8-phosphatidylethanolamine-conjugated autophagosomes [42]. Autophagy plays important roles in plant development, reproduction, starvation, environmental stress, and senescence [35,43]. Three core autophagic pathways for the degradation of chloroplasts or chloroplast proteins have been identified in plants (Figure 2): the Rubisco-containing body (RCB) pathway [44,45,46,47,48], the ATI1-GFP Labels Plastid-Associated Body (ATI-PS) pathway [49], and the whole-chloroplast autophagy (Chlorophagy) pathway [50,51].

#### 3.1.1. RCB Pathway

The initial evidence for the RCB pathway emerged from the observation of Rubisco within small spherical bodies enclosed by autophagosome-like double membranes in both the cytoplasm and vacuole of wheat leaves, which were subsequently designated as RCBs [44]. RCBs are commonly detected in senescent plant leaves, and their formation can also be triggered by premature senescence inducers such as nutrient starvation, environmental stress, and pathogen infection [46,52,53]. In Arabidopsis autophagy mutants, RCB formation is impaired, and autophagy-related proteins have been shown to interact with chloroplast protrusions and stromal tubules, confirming that RCBs are degraded via the autophagic pathway [45,46,54].

Further studies have revealed that the RCB pathway is regulated by leaf carbon status rather than nitrogen status, with carbon acting as a key negative regulator [47]. Exogenous or photosynthetically derived sugars suppress RCB appearance under light. Moreover, RCB abundance is higher in starch synthesis mutants but lower in starch overaccumulation mutants compared to wild-type plants. Under nitrogen deficiency, leaves accumulate carbon compounds and RCB formation is suppressed, while shading (preventing carbon accumulation) restores RCB formation. Under carbon deprivation, plants enhance RCB-mediated protein degradation to produce amino acids for respiration and energy maintenance. Consequently, the RCB pathway represents a crucial route for sustaining cellular energy demands during senescence by channeling chloroplast stromal proteins (particularly Rubisco) into amino acid metabolism [48,55,56]. Consistent with this, Izumi et al. [57] further demonstrated that autophagosome development and chloroplast segmentation proceed synchronously to mediate piecemeal chloroplast degradation. This spatiotemporally coordinated process ensures orderly RCB formation and efficient mobilization of stromal proteins, thereby providing a structural and mechanistic basis for robust nitrogen remobilization during leaf senescence.

#### 3.1.2. ATI-PS Pathway

ATI-PS pathway is an ATI-mediated, ATG8-dependent, stress-induced selective autophagy pathway in plants [58]. It is primarily activated under conditions such as carbon starvation, salt stress, and dark treatment, and is responsible for delivering chloroplast proteins to the vacuole via piecemeal chlorophagy, thereby maintaining chloroplast homeostasis, enabling carbon-nitrogen reuse, and promoting stress tolerance [49,59]. ATI proteins are single-pass transmembrane proteins anchored at the chloroplast outer membrane, where they directly interact with ATG8 through an ATG8-interacting motif (AIM). Upon nutrient starvation, ATI1 can assemble into two types of bodies: one mediates ER-phagy (the ATI-ER pathway), and the other forms on the chloroplast surface as ATI-PS bodies, which are ultimately delivered to the central vacuole for cargo degradation [58,59]. Notably, ATI-PS bodies differ from classical RCBs in morphology, size, and substrate specificity. Loss of ATI1 does not affect Rubisco stability but leads to significant accumulation of chloroplastic peroxiredoxin A (PrxA), indicating that ATI1 acts as a specific receptor for selective turnover of particular chloroplast proteins [58]. Functionally, Arabidopsis plants overexpressing *ATI1* exhibit enhanced tolerance to carbon starvation, whereas *ATI1* suppression results in opposite phenotypes [59]. Additionally, ATI1 and ATI2 co-mediate reticulophagy, further highlighting the multifunctional roles of the ATI family in maintaining endomembrane system homeostasis [60]. Based on current evidence, the formation and release of ATI-PS bodies from the chloroplast surface do not require ATG proteins, whereas their subsequent vacuolar delivery is ATG-dependent [58]. However, how ATI1 localized on the outer membrane perceives and takes up target proteins from inside the chloroplast remains to be elucidated.

#### 3.1.3. Chlorophagy

Unlike the RCB and ATI-PS pathways, which transport only part of the chloroplast components, chlorophagy is a selective autophagic process that engulfs entire damaged or senescent chloroplasts into autophagosomes and delivers them to the vacuole for complete degradation [18,35,61]. Upon UV-B exposure, chloroplasts accumulate substantial amounts of ROS, triggering the capture of entire chloroplasts by ATG8-labeled autophagosomes and their subsequent vacuolar degradation [50]. However, studies in the *fc2* mutant have revealed that chloroplast degradation does not require core autophagy components such as ATG5 and ATG7, suggesting the existence of alternative routes [62].

Recent evidence indicates that chlorophagy can also occur via microautophagy, in which the vacuolar membrane directly invaginates to engulf damaged chloroplasts without forming canonical double-membrane autophagosomes [51]. Lee et al. [41] further showed that this microautophagic pathway requires the ubiquitin-binding autophagy receptor NBR1 but operates independently of ATG7, thus bypassing the need for ATG8 lipidation to mediate vacuolar degradation. In this process, NBR1 coats both the surface and interior of chloroplasts (including association with thylakoids and stroma), followed by their direct engulfment into the central vacuole. The ability of NBR1 to distribute inside chloroplasts challenges the conventional view that autophagy receptors are restricted to organelle surfaces. This NBR1-mediated microautophagy is particularly activated under low temperature and high light stress, and depends on NBR1′s ubiquitin-binding capacity but not on canonical ATG proteins [41,63]. Collectively, macroautophagy constitutes the primary route for eliminating damaged chloroplasts, whereas NBR1-mediated microautophagy acts only on a relatively small fraction of chloroplasts and proteins that cannot be degraded via the CHLORAD pathway or canonical autophagy.

### 3.2. Regulation of Autophagic Activity: TOR-SnRK1 Signal Axis

**Figure 3:**
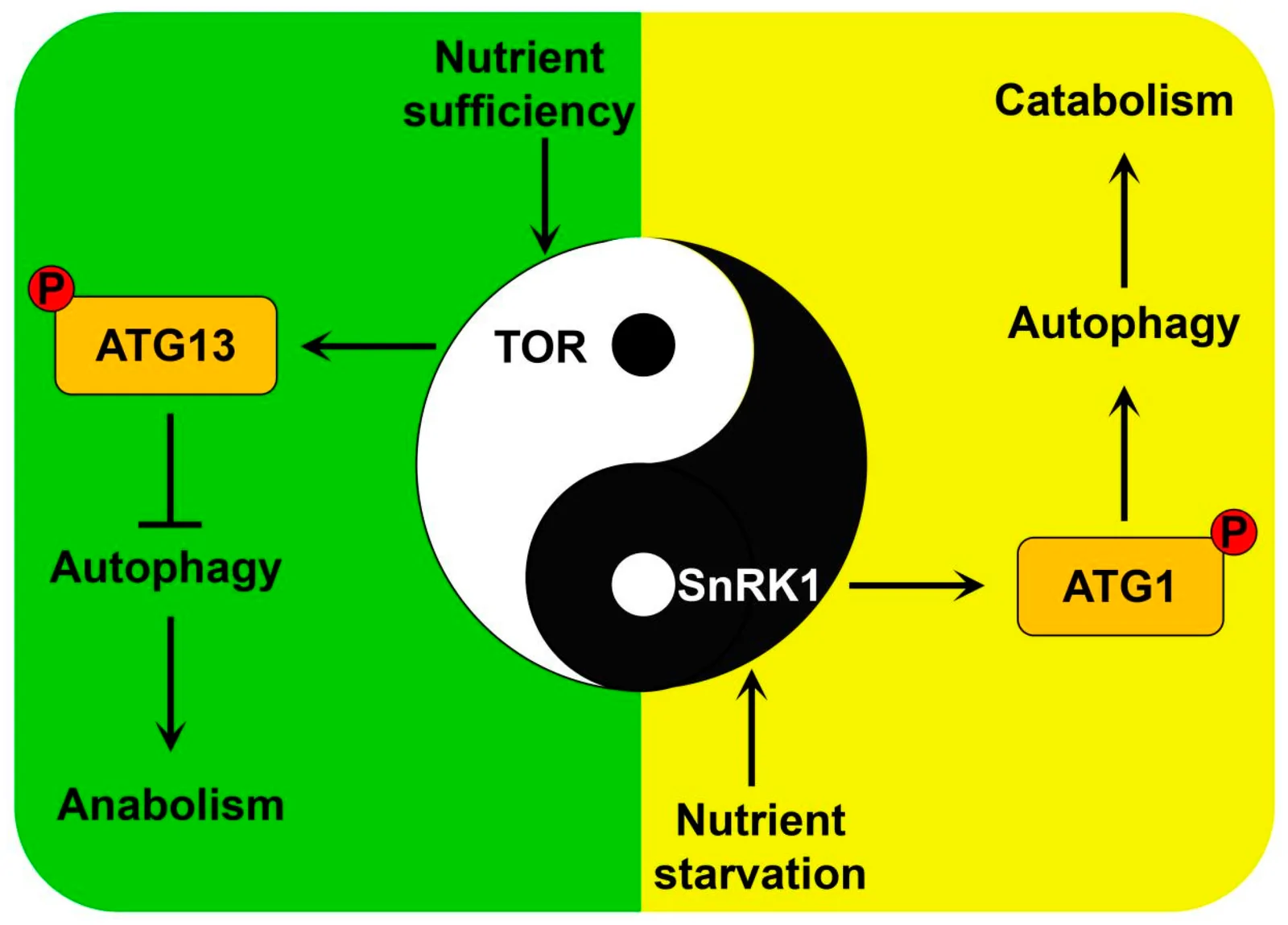
Summary of plant growth and senescence regulated by the TOR and SnRK1 kinases through autophagy in response to nutrient conditions. SnRK1 and TOR kinases act in an antagonistic manner to balance cell anabolism and catabolism, with SnRK1 promoting catabolic processes under energy stress and TOR promoting anabolic processes under nutrient sufficiency. The green part on the left in the figure represents young leaves, while the yellow part on the right represents senescent leaves. TOR, target of rapamycin; SnRK1, Snf1-related kinase1; ATG, autophagy-related protein.

Autophagic activity is finely regulated by an intracellular nutrient and energy signaling network, in which the target of rapamycin (TOR) and Snf1-related protein kinase 1 (SnRK1) play a central role [64]. TOR is a negative regulator of autophagy, SnRK1 is a positive regulator, and the two integrate carbon/nitrogen metabolism and sugar signaling to coordinately regulate autophagy and nitrogen recycling (Figure 3).

#### 3.2.1. TOR Signaling Pathway

TOR is an atypical Ser/Thr kinase, a conserved central growth regulator governing diverse anabolic processes [65,66,67]. The TOR-ATG13-ATG1 regulatory module is evolutionarily conserved from yeast to plants: in yeast, TORC1 suppresses autophagy by maintaining ATG13 phosphorylation, whereas TOR inactivation triggers ATG13 dephosphorylation and ATG1 activation [68,69]. In plants, TOR similarly acts as a negative regulator of autophagy [66,70]. Overexpressing *TOR* inhibits autophagy in Arabidopsis, while *TOR* disruption constitutively activates it [71,72]. Plants lacking ATG1 kinase complex components including ATG1, ATG13, or ATG11 are hypersensitive to nutrient deprivation and senesce prematurely due to suppressed autophagy, indicating that these components play indispensable roles in plant autophagy [73,74,75]. The Arabidopsis ATG13 protein contains a conserved TOR-signaling (TOS) motif. TOR directly phosphorylates ATG13 via the TOS motif, and this phosphorylation is blocked upon TOR inactivation [76]. Conversely, nutrient deprivation triggers the type one protein phosphatase (TOPP) to dephosphorylate ATG13, which promotes ATG1-ATG13 complex formation and stimulates ATG1 phosphorylation, thereby activating autophagy [77]. Collectively, these findings demonstrate that TOR-dependent hyperphosphorylation of ATG13 represses autophagy, while TOR inactivation relieves this inhibition to induce autophagy.

Deprost et al. [78] provided the first genetic evidence in Arabidopsis that TOR acts as a negative regulator of leaf senescence by integrating growth signals and stress responses. RNAi-mediated downregulation of *TOR* leads to a premature senescence phenotype (chlorosis and chlorophyll loss), while overexpression of *TOR* promotes growth and delays age-dependent senescence. A similar phenomenon was observed in cucumber, where increasing TOR activity/expression delayed dark-induced leaf senescence, whereas reducing TOR activity/expression accelerated [79]. Notably, plants lacking ATG13 senesce early and are hypersensitive to nutrient deprivation [73]. This paradox may reflect that basal autophagy acts primarily in quality control, and its complete loss triggers compensatory oxidative damage, leading to premature senescence.

#### 3.2.2. SnRK1 Signaling Pathway

The SnRK1 signaling pathway engages in cross-talk with TOR to coordinately regulate autophagy [64,80]. As a positive regulator of autophagy, SnRK1 can be activated by senescence-inducing environmental cues such as darkness and nutrient starvation [81,82,83]. Suppression of *SnRK1* expression leads to defects in starch accumulation, abnormal sucrose accumulation, and premature senescence [84,85], while overexpression of *SnRK1* delays senescence [86,87]. SnRK1 may couple autophagic flux with anabolic reprogramming, enabling recycling to sustain viability. Similar to the autophagy-dependent RCB pathway, SnRK1 also responds to changes in cellular carbohydrate levels and regulates carbohydrate and starch metabolism [81,82].

Under short-term carbon starvation or nitrogen deprivation, SnRK1 activates the ATG1 kinase complex while repressing TOR to promote autophagy, whereas under prolonged carbon starvation, SnRK1 activates autophagy via an alternative route that bypasses ATG1, likely through direct phosphorylation of the phosphatidylinositol-3-phosphate kinase (PI3K) subunit ATG6 by the catalytic KIN10 subunit of SnRK1 [75]. During nutrient and sugar deficiency, SnRK1 is activated while TOR is inhibited, forming a dynamic balance that lies at the core of plant energy sensing networks [88]. TOR and SnRK1 coordinate antagonistic metabolic programs to balance plant growth and stress responses [89]. SnRK1 can directly phosphorylate and inhibit some key enzymes involved in carbon-nitrogen metabolism, and regulate downstream gene expression by phosphorylating transcription factors such as bZIP63, thereby participating in the regulation of leaf senescence [90]. Moreover, SnRK1 can phosphorylate the plant-specific ESCRT component FREE1 to regulate autophagosome closure efficiency, further enhancing autophagic flux in response to nutrient stress [91]. Recent studies have identified FLZ (FCS-like zinc finger) proteins as critical integrators between SnRK1 and TOR signaling. Upon energy stress, FLZ proteins are rapidly degraded via ATG8-mediated autophagy, relieving SnRK1 inhibition and establishing a positive feedback loop to fine-tune autophagic activity [64]. This “ATG8-FLZ-SnRK1 regulatory axis” adds a new layer to the antagonistic TOR-SnRK1 network.

### 3.3. Autophagy-Independent Vacuolar Pathways

Autophagy-deficient mutants such as *atg5* and *atg7* exhibit accelerated loss of chloroplast proteins and premature senescence under both dark-induced and natural senescence conditions, indicating that autophagy-independent pathways participate in chloroplast turnover during senescence [92,93]. Two autophagy-independent pathways have been identified in recent years: the senescence-associated vacuole (SAV) pathway [94] and the chloroplast vesiculation (CV) pathway [95] (Figure 2). SAVs are small acidic membrane vesicles of non-vacuolar origin localized in the cytoplasm, possessing strong cysteine protease activity [96]. As leaf senescence progresses, cysteine protease activity gradually increases, with the most typical example being the senescence marker SAG12. Fluorescence co-localization revealed that SAG12 is localized in SAVs and exhibits high activity [96]. Subsequent studies showed that SAVs contain stromal proteins including Rubisco and glutamine synthetase 2 (GS2), but no thylakoid proteins [97]. Further research indicated that during dark-induced leaf senescence, the expression of *SAG12*, the number of SAVs, and the content of the chloroplast protein Rubisco are significantly correlated, suggesting that Rubisco is partially degraded via the SAV pathway [94]. SAVs possess very strong proteolytic activity, and sequestered chloroplast particles are rapidly degraded within SAVs [23]. However, a recent study found that SAVs not only degrade stromal proteins but may also participate in the degradation of photosystem I (PSI) proteins and chlorophyll [98]. To date, there is no evidence that SAVs use any autophagic machinery, and further investigation is needed to elucidate the molecular mechanisms by which substrates such as Rubisco are sorted and packaged into SAVs.

The key component of the CHLOROPLAST VESICULATION (CV) pathway, the CV protein, is localized to the chloroplast envelope and thylakoid membranes [95]. CV interacts with thylakoid proteins via its C-terminal domain, forming CV-containing vesicles (CCVs) that mediate the delivery of chloroplast proteins to the vacuole for degradation [95]. This process is independent of autophagy and the SAV pathway. Induction of *CV* expression accelerates leaf senescence in both Arabidopsis and tomato, while *CV* knockout postpones senescence and even improves tomato fruit quality, suggesting a conserved role of CV in the modulation of stress-induced chloroplast degradation [95,99]. In addition, the CV pathway is functionally conserved in crops such as rice, where it regulates the rate of chloroplast degradation and affects nitrogen assimilation efficiency under drought stress [100]. The SAV and CV pathways are autophagy-independent vacuolar degradation routes for chloroplast proteins, functioning in parallel with autophagic pathways to constitute an integrated regulatory network of chloroplast protein degradation.

## 4. Intra-Chloroplast Protease Systems

Chloroplasts possess an abundant resident protease system. Most proteases within the chloroplast belong to the serine, aspartic, cysteine, or metalloprotease family, among which filamentation temperature-sensitive H (FtsH), caseinolytic protease (Clp), degradation of periplasmic proteins protease (Deg), and chloroplast nucleoid DNA-binding protein of 41 kDa (CND41) are typical representatives involved in chloroplast protein quality control and senescence-associated degradation [20,21,101]. Chloroplast proteases not only execute basal quality surveillance but also deeply participate in the initiation of leaf senescence, chloroplast disassembly, and nitrogen remobilization through signaling feedback, substrate expansion, and stress cross-regulation [18,101].

### 4.1. Clp

Clp protease is an ATP-dependent serine protease composed of a fourteen-subunit proteolytic core complex and chaperone subunits, and is primarily localized to chloroplast stroma and inner envelope membrane [102,103]. The core function of the Clp system is quality control of stromal proteins, responsible for removing improperly folded, aberrantly cleaved, or damaged stromal proteins during translocation, thereby maintaining chloroplast protein homeostasis and photosynthetic complex assembly [103,104]. During leaf senescence, the expression of numerous Clp-related subunits, including ClpD, ClpP3 and ClpP5, is significantly upregulated, which may be associated with the bulk degradation of stromal proteins [8,103,105,106,107]. The *clpc1-1* null mutant shows severe premature senescence and persistent pale leaves, demonstrating that loss of Clp function directly accelerates senescence. Based on mRNA co-expression network analysis, Rei Liao et al. [108] revealed that the core subunits of the chloroplast Clp protease system (ClpPRT, ClpD, and ClpF) are directly involved in the recognition of senescent chloroplasts, protein degradation, and organelle clearance. Loss of Clp leads to abnormal chloroplast clearance, resulting in severe premature senescence. Under leaf senescence and iron deficiency conditions, the Clp system participates in plant adaptation to senescence and iron deficiency by regulating the abundance of the Fe-S cluster biosynthesis complex SUFBC2D [109]. The role of the Clp system during senescence may be more about maintaining chloroplast proteome homeostasis, thereby creating conditions for orderly degradation. These findings extend the function of the Clp system from basal protein quality control to dynamic remodeling of specific metabolic complexes during senescence.

### 4.2. FtsH

FtsH is an ATP-dependent metalloprotease, which is localized to the thylakoid membrane [20,21]. The FtsH complex is a heterohexamer composed of FtsH1, FtsH2, FtsH5, and FtsH8 subunits [110]. Its degradation targets include key thylakoid components such as the inactive photosystem II core subunit D1 and light-harvesting pigment-protein complexes [21,111]. In addition, FtsH has been proposed to have dual roles, with FtsH2 and FtsH5 also participating in the assembly of the PSI complex, although the precise mechanism remains elusive [112]. FtsH2 encoded by *VAR2* plays an irreplaceable role in PSII protein homeostasis, as its null mutant exhibits variegated leaves and decreased photosynthetic efficiency at normal temperatures [113,114], and the variegation induced by FtsH2 deficiency is probably independent of D1 degradation [115]. A recent study revealed cross-regulation between the chloroplast internal protease system and the autophagic pathway [116]. FtsH2 interacts with the key autophagy protein ATG8a to coordinately regulate chloroplast division and degradation, thereby enabling efficient nutrient remobilization. This direct physical interaction between a chloroplast-resident protease and an autophagy core protein provides the first molecular evidence for crosstalk between intra-organellar proteolysis and vacuolar degradation.

### 4.3. Deg

D1 degradation is accomplished by the cooperative action of FtsH and Deg proteases [117,118]. Deg proteases belong to the Deg/HtrA family, a group of ATP-independent serine proteases conserved across almost all domains of life [119]. Arabidopsis chloroplasts harbor at least five Deg proteases, among which Deg1, Deg5, and Deg8 are located in the thylakoid lumen, Deg2 on the stromal side of the thylakoid membrane, and Deg7 in the stroma. All Deg isoforms exhibit intrinsic proteolytic activity except for Deg5 [21]. Deg1 defect causes inefficient PSII assembly [120]. Deg1 and Deg2 mediate the degradation of light-harvesting proteins, including light-harvesting complex II (LHCII) antenna protein subunits under environmental stresses [121,122]. Deg2 is required for normal plant development, as *deg2* mutants exhibit reduced leaf area, altered chloroplast ultrastructure, and a lack of early senescence features such as undulation of the chloroplast envelope and thylakoids [122]. Deg5 and Deg8 form a hexamer that cooperatively degrades the photodamaged D1 protein of PSII [117,123]. However, Itafa et al. [124] found that lack of Deg5 and Deg8 in tomato does not impair photosynthetic performance, contrasting with the high-light sensitivity and defective D1 degradation of the Arabidopsis *deg5 deg8* double mutant, suggesting that Deg5/Deg8 functions in PSII repair are not universally conserved. Deg7 directly participates in PSII repair by primary cleavage of photodamaged D1, D2, CP47, and CP43 [125]. The critical role of Deg7 in photoprotection is further supported by evidence from wheat, where silencing *TaDeg7* leads to reduced photosynthetic efficiency and increased sensitivity to high-light stress [126].

### 4.4. CND41

CND41 is a chloroplast-localized aspartyl protease with DNA-binding activity [127,128]. In vitro, CND41 exhibits strong proteolytic activity under acidic conditions (pH 2–4) [129] and can degrade denatured Rubisco under physiological conditions [128]. Antisense tobacco plants with reduced CND41 levels maintain green leaves and higher Rubisco content during senescence, whereas constitutive expression of *CND41* accelerates senescence and promotes Rubisco degradation [128,130]. During leaf maturation, CND41 undergoes post-translational processing from its full-length form to a shorter fragment, a step essential for its proteolytic activity and the onset of senescence [130]. Kato et al. speculated that DNA-binding might regulate its proteolytic activity [128], but the precise in vivo activation mechanism remains elusive.

A possible explanation is that CND41 may undergo acid activation in SAVs or the central vacuole, and its translocation might involve SAV and autophagy pathways [3]. Although CND41 has been characterized mainly in tobacco, functional conservation is supported by emerging evidence: a CND41-like protease is upregulated during low-nitrogen-induced senescence in Arabidopsis [131], and the *cnd41* ortholog is strongly induced in barley leaves under carbohydrate-accelerated senescence [132]. These findings collectively implicate CND41 in Rubisco turnover and nitrogen remobilization during leaf senescence, although direct evidence for the proposed activation model is still lacking.

## 5. Coordination of Proteolytic Pathways During Leaf Senescence

Chloroplast proteins account for more than 70% of total leaf protein and represent the primary nitrogen reservoir in plants, with Rubisco being the largest natural nitrogen pool [3]. The orderly degradation of chloroplast proteins is a central step in the release of nitrogen from senescent leaves and its transport to young leaves, grains, and storage organs for recycling, directly determining plant nitrogen use efficiency (NUE) and nitrogen remobilization efficiency (NRE), and ultimately affecting crop yield and quality [1,3].

The three major proteolytic systems, including the UPS, vacuolar degradation (autophagy-dependent and -independent), and intra-chloroplast proteases, do not operate in isolation. Based on current evidence, a conceptual temporal coordination can be envisioned: the UPS, particularly the SP1-CHLORAD pathway, may act early to limit the import of newly synthesized photosynthetic proteins into chloroplasts [27,29]. As senescence progresses, autophagy becomes the dominant route for bulk nitrogen recovery, delivering RCBs, ATI-PS bodies and even entire chloroplasts to the vacuole [45,46,50]. Later, intra-chloroplast proteases (Clp, FtsH, Deg) and vacuolar-independent pathways (SAV, CV) fine-tune the degradation of residual proteins and contribute to local amino acid recycling [21,95,128]. However, this sequential view is a simplification, as these pathways are likely to overlap in time and vary in relative importance depending on species, developmental stage, and environmental conditions. For example, autophagy can be activated before visible senescence under carbon starvation, and intra-chloroplast proteases operate continuously in protein quality control throughout the chloroplast lifecycle [18,47,77].

The TOR-SnRK1 nutrient-sensing network plays a central role in orchestrating autophagic activity [64]. Under carbon or nitrogen sufficiency, TOR suppresses autophagy; upon starvation, TOR inactivation and SnRK1 activation relieve this inhibition, promoting autophagic flux and nitrogen export from senescing leaves. Emerging evidence reveals direct interactions between chloroplast-resident proteases and the autophagic machinery. For example, FtsH2 interacts with ATG8a to coordinate chloroplast division and degradation [116]. Moreover, ubiquitinated TOC components can be diverted either to the 26S proteasome (via K48-linked chains) or to selective autophagy (via K63-linked chains recognized by NBR1), illustrating a molecular switch between the UPS and autophagy [32].

## 6. From Mechanisms to Crop NUE: Translating Chloroplast Proteolysis into Agronomic Gains

The coordinated proteolytic network offers multiple intervention points for improving NUE in crops. Table 1 summarizes the key features of each pathway and the current evidence base for its relevance to NUE. Below, we discuss how each can be manipulated, focusing on strategies that have already shown promise in model species or that are most likely to succeed in crops.

### 6.1. Enhancing Autophagy for Direct Nitrogen Recovery

As the major route for bulk nitrogen export in plants, autophagy is a prime target for improving NUE. Multiple mutant studies have validated its critical function in nitrogen recycling. Arabidopsis *atg5-1* and *atg7-1* mutants are defective in nitrogen reuse [92]. Under low nitrogen, autophagy contributes up to 60% of leaf-to-seed nitrogen transport, and *atg5* mutant retains abundant nitrogenous substances in senescent leaves [133]. In maize and rice, impaired autophagy disrupts Rubisco turnover and nitrogen remobilization, leading to inhibited growth, accelerated senescence and reduced yield [134,137]. Genetic manipulation of autophagy has achieved favorable agronomic performance. Overexpression of soybean *GmATG8* improves low-nitrogen tolerance and yield in Arabidopsis [138]. Moreover, overexpression of core autophagy genes *ATG7* or *ATG8* also enhances nitrogen remobilization and grain yield in Arabidopsis and rice [135,137,139]. Notably, constitutive hyperactivation of autophagy accelerates premature leaf senescence [136]. To bypass this detrimental side effect, senescence-inducible promoters such as *SAG12* have been deployed to confine enhanced autophagic flux specifically to senescent leaves via spatiotemporal transcriptional control [140].

### 6.2. Modulating Senescence Timing via UPS and Vacuolar-Independent Pathways

The UPS, through SP1, regulates the timing of chloroplast protein import [28]. Moderate downregulation of SP1 specifically during senescence preserves the import of photosynthetic proteins in early senescent leaves, thereby extending the functional photosynthetic phase [141]. Similarly, genetic disruption of *CV* evidently delays leaf senescence, as demonstrated in Arabidopsis [95], rice [100], soybean [142] and tomato [99]. In comparison, the molecular mechanism of SAV pathway remains incompletely understood, and no effective genetic tools have yet been established to manipulate this pathway for agricultural applications.

### 6.3. Fine-Tuning Intra-Chloroplast Proteolysis

Intra-chloroplast proteases primarily maintain photosystem function by removing damaged proteins. Their contribution to whole-plant nitrogen recycling is indirect but significant for stress tolerance. For example, the interaction between FtsH2 and ATG8a [116] suggests that coordinating protease activity with autophagy could improve both protein quality control and nitrogen remobilization. However, functional redundancy among the >20 chloroplast protease families makes single-gene manipulation unpredictable. A systems approach, possibly through modulating upstream regulators, will be required to harness their full potential.

### 6.4. Exploiting the TOR-SnRK1 Signaling Hub

TOR suppresses autophagy while SnRK1 activates it. This central regulatory point can be targeted to induce autophagy independently of nutrient status. Chemical inhibition of TOR (e.g., with rapamycin analogs) or genetic knockdown of *TOR* using RNAi triggers autophagy even under nitrogen sufficiency, potentially boosting nitrogen remobilization. Conversely, overexpressing *SnRK1* or its downstream transcription factor *bZIP63* delays senescence under energy stress [90]. Since TOR and SnRK1 control numerous growth processes, any manipulation must be confined to senescing tissues using tissue-specific promoters to avoid pleiotropic effects.

### 6.5. Translational Challenges

**Figure 4:**
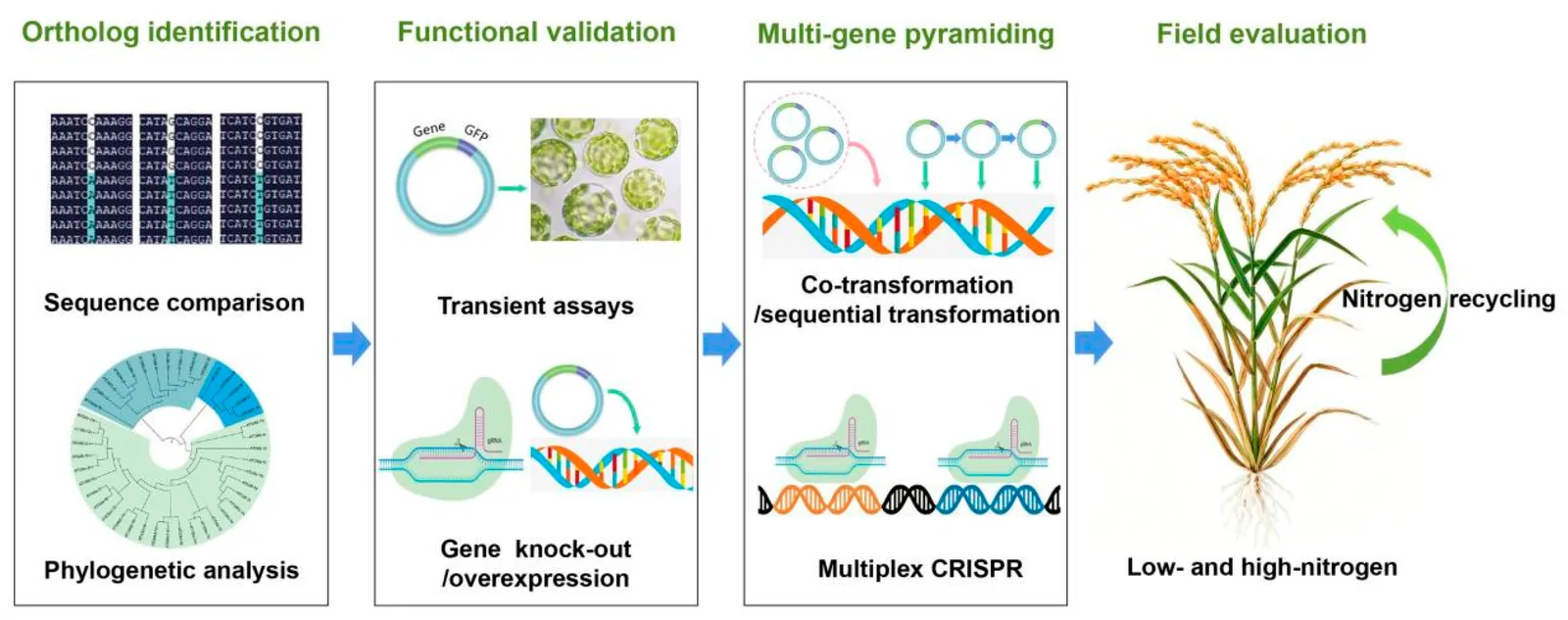
Proposed framework for genetic improvement of NUE in crops. Orthologs of validated regulators are identified in target crops by sequence comparison and phylogenetic analysis. Their functions in senescence-associated nitrogen recycling are validated using transient assays or stable knockout/overexpression lines. Complementary genetic modifications are then pyramided into a single elite background via co-transformation, sequential transformation, or multiplex CRISPR. Finally, NUE assessment is performed under contrasting nitrogen levels in field trials.

The three chloroplast protein degradation systems (the UPS, vacuolar pathways, and intra-chloroplast proteases) are evolutionarily conserved [20,30,67,100,143], yet their relative contributions to nitrogen remobilization likely diverge substantially among crop species. Translating this mechanistic knowledge into improved NUE faces three major bottlenecks: (1) functional conservation of the identified regulators (e.g., SP1, ATG8, CV, Clp, FtsH) in crops remains largely untested; (2) the synergistic or antagonistic effects of simultaneously manipulating multiple pathways are unknown; and (3) field validation under fluctuating nitrogen supply demands carefully standardized experimental designs. Given that these core genes have already been functionally validated in model plants (and in some cases crops), we propose a direct, function-driven pipeline (Figure 4) that bypasses natural variation screening or GWAS:

(1) Ortholog identification: Identify orthologs of the validated genes in target crops using sequence similarity searches and phylogenetic analysis.
(2) Rapid functional validation: Confirm that the orthologs control senescence-associated nitrogen recycling by transient assays or stable knock-out/overexpression lines.
(3) Multi-gene pyramiding: Stack several complementary genetic modifications into a single elite background via co-transformation, sequential transformation, or multiplex CRISPR.
(4) Multi-location field trials: Evaluate the pyramided lines under low- and high-nitrogen regimes across multiple environments. Key traits include grain yield, NRE, NUE, and senescence dynamics.

## 7. Conclusions and Perspectives

The coordinated degradation of chloroplast proteins by the UPS, vacuolar pathways, and intra-chloroplast proteases is not a linear cascade but a spatiotemporally integrated network that enables efficient nitrogen remobilization during leaf senescence. The TOR-SnRK1 nutrient-sensing network acts as a master rheostat, balancing autophagic flux against cellular energy status. Despite this broad framework, several fundamental questions remain unresolved.

First, what signals govern the transition from UPS-mediated quality control to autophagy-driven bulk degradation? Second, what are the mechanistic details of microautophagy and autophagy-independent vacuolar pathways, particularly cargo selection and vesicle formation? Third, how are the sequential waves of proteolytic activity switched on and off without causing metabolic chaos? Fourth, can we genetically uncouple the beneficial nitrogen-recycling function of autophagy from its potential to accelerate premature senescence? Emerging evidence of non-canonical pathways (e.g., NBR1-mediated microautophagy bypassing ATG8 lipidation) and direct crosstalk between chloroplast proteases (FtsH2) and the autophagic machinery (ATG8a) suggests a far more integrated web than previously appreciated. Unraveling how this web is wired and how it can be rewired for crop improvement remains a central challenge for the field.

## Figures and Tables

**Table 1:** Comparative analysis of chloroplast proteolytic pathways during leaf senescence.

Pathway	Primary Cargo	Substrate Selectivity	Effect on Senescence Phenotype	NUE Relevance	Key References
UPS (SP1/PUB4)	TOC translocon components; ubiquitinated outer membrane proteins	High	SP1 loss promotes protein import; SP1 overexpression reduces import	Emerging (mainly in Arabidopsis)	[27,29,30,34]
Autophagy-dependent (RCB/ATI-PS/chlorophagy)	Stromal proteins (Rubisco), plastid proteins, entire chloroplasts	Selective	Both ATG loss and hyper-autophagy may accelerate senescence; moderate autophagy prevent premature senescence	Strong (validated in Arabidopsis, rice, maize)	[45,46,47,48,50,58,132,133,134,135,136]
Autophagy-independent (SAV/CV)	Stromal proteins; thylakoid proteins	Selective	CV knockout delays senescence	Moderate (conserved in rice, soybean, tomato)	[94,95,99,100]
Intra-chloroplast proteases (Clp/FtsH/Deg/CND41)	Misfolded, photodamaged, or incompletely processed thylakoid/stromal proteins	High	Clp deficiency causes severe premature senescence	Moderate (primarily quality control)	[21,101,102,105,106,109,128]

## Data Availability

No new data were created or analyzed in this study. Data sharing is not applicable to this article.
